# Supplementary material for: The genome sequence of the Common Mummy Wasp, Aleiodes similis (Curtis, 1834) (Hymenoptera: Braconidae)
Source: Wellcome Open Res. 2026 Mar 18;11:169. [Version 1] doi: 10.12688/wellcomeopenres.26127.1 (PMC13049424; doi:10.12688/wellcomeopenres.26127.1)
Supplement: Peer review history

# Reviewer report: Reviewer response for version 1

**DOI:** 10.21956/wellcomeopenres.28774.r150995

**Published:** 2026-04-03

**Authors:** Marta Coronado-Zamora, Vicente Chillida

**Affiliations:** 1 Autonomous University of Barcelona Department of Genetics and Microbiology (Ringgold ID: 196650), Bellaterra, Catalonia, Spain

This data note describes the first high quality chromosomal assembly of the Common Mummy Wasp, *Aleiodes similis*. The genome data are available at NCBI BioProject, and data is accessible as checked in March 2026.  The genome assembly is highly contiguous, with overall very good assembly metrics. The mitochondrial genome is also included. The genome currently lacks gene annotations, although RNA-seq was also produced.

**Comments:**

- Regarding the RNA extraction, although the text states the use of Nanodrop, Qubit, and Agilent RNA 6000 Pico kit, neither the RNA concentration nor any RNA integrity metrics are reported. Nevertheless, for DNA extraction, quality metrics are provided.
- Regarding haplotype 2, why it was assembled to the scaffold level while the haplotype 1 was able to be assembled to the chromosome level? Also, haplotype 2 has better statistics for N50 contig and scaffold values.
- The other specimen collected for RNA-sequencing was also a female?
- The sentence “For haplotype 1, the estimated QV is 64.6, and for haplotype 2, 64.6. When the two haplotypes are combined, the assembly achieves an estimated QV of 64.6.” is highly repetitive, it could be rephrased.
- The EBP metric is not explained, it would be useful to explain how to interpret this value.
- There are some software cited in Table 5 but not mentioned in the main text: Cooler, fasta_windows and GoaT CLI.

Are sufficient details of methods and materials provided to allow replication by others?

Yes

Is the rationale for creating the dataset(s) clearly described?

Yes

Are the datasets clearly presented in a useable and accessible format?

Yes

Are the protocols appropriate and is the work technically sound?

Yes

Reviewer Expertise:

genomics, bioinformatics, population genetics, adaptation

We confirm that we have read this submission and believe that we have an appropriate level of expertise to confirm that it is of an acceptable scientific standard.

# Reviewer report: Reviewer response for version 1

**DOI:** 10.21956/wellcomeopenres.28774.r150991

**Published:** 2026-03-28

**Authors:** Lindsey C Perkin

**Affiliations:** 1 USDA, Agricultural Research Service, College Station, Texas, USA

Summary: The paper describes the genome assembly of the common mummy wasp, *Aleiodes similis*. The author chose this insect because it is a parasitoid of a range of Noctuidae caterpillars and hard to distinguish morphologically with *A. gastritor*. The author clearly describes and link the protocols and materials and methods for the assembly to repeated. 

 The datasets are useable and put into depositories. I only have a couple edits and suggestions. 

 1) In the Background section.

 The first sentence in paragraph two should read "..i.e., it has MORE than one generation per year."

 2) RNA is extracted and sequenced but doesn't seem to be used. Could you do gene annotations?

Are sufficient details of methods and materials provided to allow replication by others?

Yes

Is the rationale for creating the dataset(s) clearly described?

Partly

Are the datasets clearly presented in a useable and accessible format?

Yes

Are the protocols appropriate and is the work technically sound?

Yes

Reviewer Expertise:

gene expression, genomics, molecular biology

I confirm that I have read this submission and believe that I have an appropriate level of expertise to confirm that it is of an acceptable scientific standard.
